# Editorial Announcement

**Published:** 1913-08

**Authors:** 


### Editorial Announcement

The Buffalo Medical Journal will co-operate with any medical organization or any organization of an allied profession or representing any public movement in which physicians are especially interested by establishing a monthly or quarterly department, under the editorial management of such organization. Any such department that may be added will be supplementary to the present form of the Journal, and the charge made will be based on actual increase of expense. Except as to the expediency of establishing and maintaining such department, its management may be entirely independent and an adequate announcement will be made on the Cover and in the Table of Contents.

